# P53/NANOG balance; the leading switch between poorly to well differentiated status in liver cancer cells

**DOI:** 10.3389/fonc.2024.1377761

**Published:** 2024-05-23

**Authors:** Fazeleh Ranjbar-Niavol, Niloufar Rezaei, Ying Zhao, Hamed Mirzaei, Moustapha Hassan, Massoud Vosough

**Affiliations:** ^1^ Department of Regenerative Medicine, Cell Science Research Center, Royan Institute for Stem Cell Biology and Technology, Academic Center for Education, Culture and Research (ACECR), Tehran, Iran; ^2^ Experimental Cancer Medicine, Institution for Laboratory Medicine, Karolinska Institute and Karolinska University Hospital-Huddinge, Huddinge, Sweden; ^3^ Research Center for Biochemistry and Nutrition in Metabolic Diseases, Institute for Basic Sciences, Kashan University of Medical Sciences, Kashan, Iran

**Keywords:** hepatocellular carcinoma, p53, NANOG, molecular mechanism, cancer

## Abstract

Enforcing a well-differentiated state on cells requires tumor suppressor p53 activation as a key player in apoptosis induction and well differentiation. In addition, recent investigations showed a significant correlation between poorly differentiated status and higher expression of NANOG. Inducing the expression of NANOG and decreasing p53 level switch the status of liver cancer cells from well differentiated to poorly status. In this review, we highlighted p53 and NANOG cross-talk in hepatocellular carcinoma (HCC) which is regulated through mitophagy and makes it a novel molecular target to attenuate cancerous phenotype in the management of this tumor.

## P53/NANOG balance and hepatocellular carcinoma

**Figure 1:**
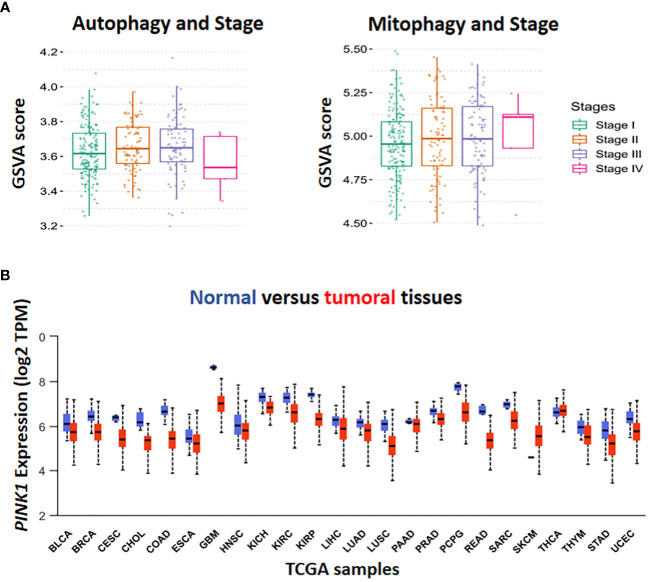
*In-silico* analysis by The Cancer Genome Atlas (TCGA) database. Gene set variation (GSVA) score has been done for the assessment of the association in autophagy, mitophagy and different stage of HCC **(A)**. The level of *PINK1* expression in tumor condition compared to normal was illustrated in TCGA boxplot cohort among the various types of cancer. Box plot display log_2_ of the Transcript per Million (TPM) values for *PINK1* expression in different types of cancer against normal ones in cohort study **(B)**.

Hepatocellular carcinoma (HCC) is the most common form of primary malignancy in the liver and a significant cause of global cancer related-death (1). HCC usually occurs in advanced liver diseases such as hepatitis B and C virus (HBV, HCV) infections (2). The major risk factors for the development of HCC include age, male gender, metabolic dysfunction associated steatosis liver disease (MASLD), type 2 diabetes mellitus (T2DM), inherited metabolic disease, obesity, and exposure to aflatoxin B1 (3). Surgery, ablation therapy, radiotherapy, systemic and targeted therapy play important treatment roles in either primary or advanced stages of hepatocellular carcinoma (4). The biological process of carcinogenesis in HCC is complex due to several factors (5). In cancer cells, autophagy has opposite roles in early and late stages of cancer (6). In the early stages of tumorigenesis, autophagy has a tumor suppressive function via preventing tumor initiation, proliferation, invasion, and metastasis (7, 8). However, during tumor development, autophagy facilitates for tumor cells to survive under stressful circumstances and appears to be a crucial factor for tumor cell metastasis (9, 10). Autophagy, as a catabolic process, enables cells to eliminate damaged and unfolded protein aggregates and degrade organelles for recycling their structural biomolecules (11). Cellular homeostasis depends on autophagy and its disruption may result in several diseases, particularly cancers (12). As shown in (**Figure 1A** Autophagy), the GSVA score of autophagy has been raised in the stages II and III because of the increasing the aggressiveness of tumor cells through promoting metastasis which is dependent on a number of variables, including genetic background, type of cancer, grade/stage of tumor, and tumor microenvironment (9). A series of experiments indicated that in early tumorigenesis, the tumor- suppressive role of autophagy changes into pro-tumorigenic function due to genetic perturbation, such as deletion of Atg7 *Atg7* in mice (13).

There is contradictory data about autophagy’s function in hepatocellular carcinoma (HCC), with some studies pointing to both tumor-promoting and tumor-suppressive effects (14). Autophagy can function as a tumor suppressor through reducing oxidative stress (15), preserving genomic stability (16), and preventing uncontrolled inflammation (17), eliminating cancer-causing proteins and inducing cell death (18), but operates as tumor promotion in later stages by boosting cancer stem cell formation, invasion and tumor metastasis (19). In addition, the expression of 3-Hydroxybutyrate Dehydrogenase 2 (*BDH2*), a factor in triggering apoptosis and suppressing autophagy, is downregulated in tumor promotion stages (14). It is also believed that the overexpression of the PD-1/PD-L1 axis in HCC leads to autophagy induction by increasing the rate of ATG13 expression and tumor proliferation (20).

Autophagy affects liver cancer development by preventing tumor suppressor activities or increasing chemoresistance in HCC cells (11). Impaired autophagy can inhibit HCC growth by activating tumor suppressors such as p53, p16, p21, and p27. Autophagy also leads to resistance to targeted therapy drugs in HCC cells, including resistance to sorafenib, the only systemic therapy approved by the FDA for HCC. Autophagy inhibition can increase the sensitivity of HCC cells to chemotherapeutic drugs and boost sorafenib’s anti-proliferative activity (21). In addition, autophagy contributes significantly to HCC drug resistance induction through some significant molecular pathways including, MAPK, TGF-β, NF-κB, Beclin 1, p62, NRF2, and non-coding RNAs (11).

Initial studies provided evidence that autophagy facilitates metastasis by activation of several signalling pathways, including migration and colonization of cancer cells (22), the induction of epithelial-mesenchymal transition (EMT) (23), detachment-induced apoptosis (anoikis) resistance (24), hypoxia and nutrient-deprived adjustment and adaption to thrive in diverse cellularmicroenvironments (25). The pro-metastatic impact of autophagy increases interest in modulating autophagy as a possible preventative and therapeutic strategy (26). In contrast (**Figure 1A**, Autophagy, stage IV), recently, several studies have illustrated the critical role of autophagy pathway in regulating dormancy emergence and metastatic suppression (27–30). Autophagy inhibition by knocking down ATG3 results in an exit from dormancy, causing the proliferation of metastatic cells and enrichment of cancer stem cells population. In other words, autophagy suppression lead to producing more aggressive subpopulations (27).

After tumor establishment, in response to stresses caused by chemotherapy, autophagy serves as a survival pathway (31). In addition, mitophagy is, a type of autophagy, a highly precise quality control mechanism that destroys malfunctioning mitochondria and increases mitochondrial turnover (32).

Inadequate function of old, damaged or dysfunctional mitochondria in oxidative stress induces tumorigenesis (33) (34). Cancer initiation is accompanied by ROS production and increased mitophagy. Moreover, It has been shown that cancer cells can obtain CSC-like properties as a result of mitochondrial dysfunction (35). Therefore, enhanced mitophagy reduces mitochondria number, which results in low reactive O_2_ concentration and declining in energy level. Finally, in hypoxic conditions, CSCs survive better and this worsen cancer status (**Figure 1A**, Mitophagy, stage IV). Although chemotherapeutic agents target rapidly proliferating and ROS-producing cells, they are inefficient against quiescent cells (e.g., CSCs),causes drug resistance (36).

**Figure 2:**
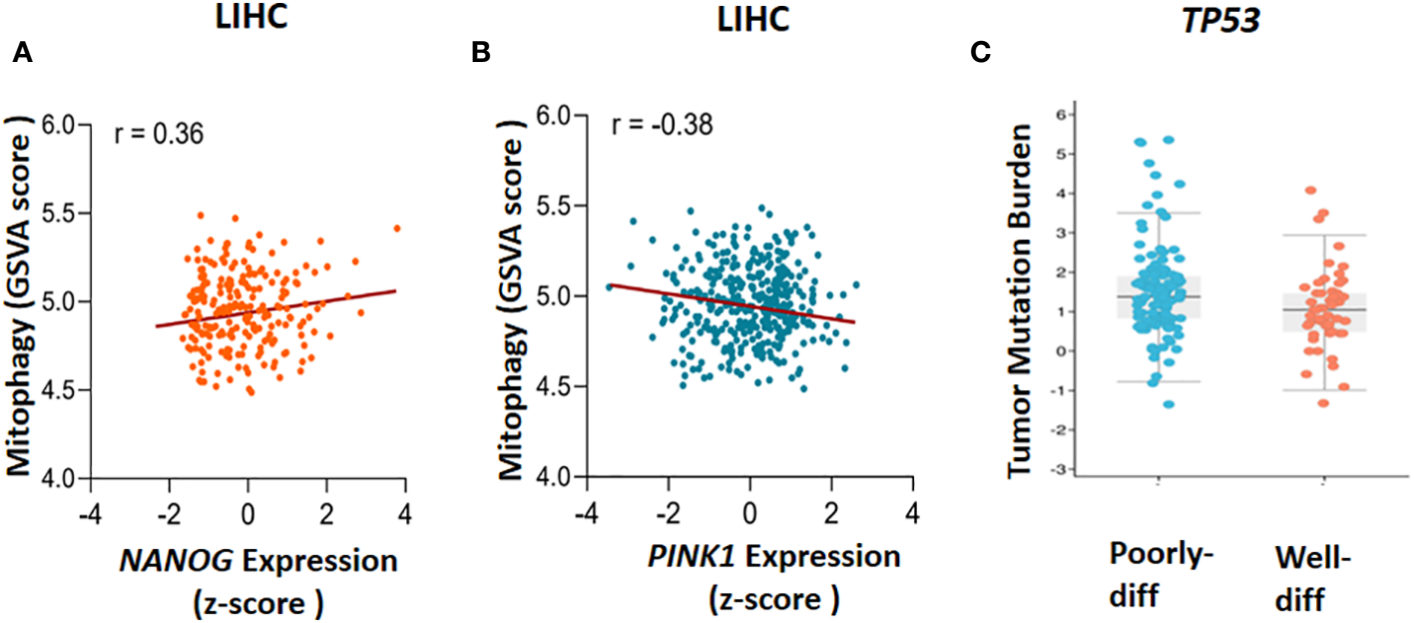
The Cancer Genome Atlas (TCGA) database analysis. The correlation between mitophagy and gene expression has been found through gene set enrichment (GSVA) score and Z-score calculation, **(A)** *NANOG* expression and mitophagy (r=0.36) and **(B)** *PINK1* expression and mitophagy (r=-0.38) has been demonstrated. To clarify the *TP53* mutation in poorly and well differentiated (poorly-diff and well-diff) state, tumor mutation burden has been done **(C)**. LIHC, liver hepatocellular carcinoma.

In 2010, Noriyuki Matsuda found that in impaired mitochondria, tensin homolog-induced putative kinase 1 (PINK1) can launch mitophagy by accumulating on the outer mitochondrial membrane (OMM). Then, Mitofusin2 (Mfn2) and other substrate like ubiquitin phosphorylated by PINK1 to recruit Parkin (encodes by *PRKN* gene) from cytoplasm to mitochondria. When Parkin is joined to OMM by PINK1, some significant ubiquitinated substrates such as Voltage-dependent anion channel 1 (VDAC1), Mfn-2 and other mitochondrial–related proteins can facilitate their interaction with Sequestosome-1 (Sqstm1/p62) that in turn stimulates interaction with LC3 to promote the engulfment of defective mitochondria (37). To validate previous investigations, the GSVA package in combination with z-score has been used to assess *PINK1* and *NANOG* expression and the calculation of gene set enrichment score in mitophagy (**Figures 2A, B**). The cyto-protective features of mitophagy initiate chemoresistance following treatment with chemotherapy (11, 38, 39). Some chemotherapeutic drugs cause malfunctioning mitochondria, generate some cytotoxic by-products like Reactive Oxygen Species (ROS), and alter regular metabolic processes (40). By preventing the build-up of dysfunctional mitochondria, which can increase ROS production and cause cell damage, mitophagy plays a critical role in preserving cell and tissue homeostasis. However, it may also speed up tumorigenesis by allowing cancerous cells to adapt to the microenvironment change (41). Given the intra-tumoral heterogenousity, cancer stem cells (CSCs) are regarded as a subset of cancerous cells that can self-renew and differentiate to generate heterogeneous sub-populations. Chemotherapy-resistant CSCs are frequently blamed for cancer recurrence. Holding stemness state and the maintenance of CSCs in hepatocarcinogenesis depends critically on mitophagy It is suggests that the targeting of mitophagy might decrease CSCs population in HCC (42).

*TP53* has been referred to as the “guardian of the genome” because of its essential function in preventing neoplastic transformation (43). This tumor suppressor is involved in the regulation of autophagy, pro-autophagic and anti-autophagic phenotypes, depending on its intracellular location (44). For many years, the direct involvement of p53 protein in the stemness regulatory network has been investigated. Protein p53 has been shown to decrease self-renewal and increase differentiation in embryonic stem cells (ESCs) and cancer cells (13). Moreover, the induction of apoptosis, or programmed cell death, is one of the most dramatic reactions to p53 activation. In hepatocytes and many other cell types, apoptosis occurs through either of two major pathways, known as the extrinsic death receptor pathway or the intrinsic mitochondrial pathway (45). In the mitochondrial pathway, death signals impact mitochondria directly or indirectly through proapoptotic Bcl-2 family members like Bak and Bax transduction. Apoptogenic proteins are subsequently released by the mitochondria, which eventually causes caspase activation and apoptosis. The death receptor pathway involves the recruitment of adaptor proteins such as initiator caspases 8 by ligand-bound receptors, which in turn activates caspases to trigger programmed cell death. Although p53-dependent apoptosis normally proceed via the mitochondrial pathway, p53 can also regulate cell death through death receptors. Moreover, the transcription of several proapoptotic genes, including those encoding Bcl-2 family members such the BH-3-only proteins Bax, Noxa, and Puma, can be activated by p53 (46). p53 mutations in hepatocellular carcinoma are frequently detected. These alterations may result in the loss of p53’s tumor-suppressive properties, which would contribute the progression and migration of hepatoma cells (47).

P53 previously showed that play an important role in EMT and invasion of liver cancer cell. Knocking down p53 in liver cancer cells increased sensitivity to insulin- and TGF-β1-induced alterations in EMT markers as E-cadherin, ZO-1, Snail, Zeb1, and vimentin. Secondly, p53 knockdown greatly increased insulin, Wnt and TGF-β1-induced migration of liver cancer cells. Finally, p53 deletion significantly increased the *in vivo* metastasis of liver cancer cells (48).

NANOG is one of the primary transcription factors responsible for the pluripotency in pluripotent and cancer stem cells (49). Enhanced cell proliferation, accelerated migration and invasion of cells, chemoresistance, adaptation to hypoxia, and immunological evasion of cancer cells are all mediated by NANOG (50). In addition, among all genes associated to maintaining stemness feature of cells, NANOG has also been found in a variety of tumor types, including oral, kidney, liver, prostate, breast, ovarian, cervix, lung, stomach, brain, and prostate malignancies. Higher expression of NANOG has been linked to poor prognosis in patients with HCC, ovarian serous carcinoma, colorectal cancer, and breast cancer (50, 51). In addition to being an overexpressed biomarker for CSCs and HCC clinical progression, NANOG is essential for sustaining the self-renewal of liver CSCs via the insulin-like growth factor-1 (IGF-1R) signaling pathway. NANOG is a biomarker that not only helps identify CSCs but also demonstrates tumorigenesis, self-renewal, ability to infiltrate and metastasis, and resistance to chemotherapy drugs like cisplatin and sufentanil. A study by Zhou et al. (52) demonstrated that downregulating of NANOG can enhance chemosensitivity while preventing hepatocellular carcinoma cells from proliferation, invasion, and migration (53).

Thus, NANOG could be a potential target for cancer therapy, in addition to prevent carcinogenesis. The emerging evidence indicated that the regulation of mitophagy in hepatocellular CSCs is mediated by cross talking of p53 and NANOG (54).

It has been shown that the regulation of a wide range of cellular events including, autophagy, ferroptosis, cell cycle arrest and senescence, genomic integrity, DNA damage repair, apoptosis, and metabolism are all influenced by p53 (54).

In HCC, stemness maintenance is driven by enhanced mitophagy through removing p53. A PINK1-mediated mitophagy pathway phosphorylates p53 at S392, then phosphorylated p53 eliminated, resulting in increased production of NANOG, a transcription factor that enhances the self-renewal potential of liver cancer stem cells (LCSCs). In contrast, when the function of mitophagy is disrupted, phosphorylated p53 cannot be eliminated by mitophagy and is delivered to the nucleus, where it binds to the *NANOG* promoter and suppresses the production of NANOG, leading to a decrease in hepatic cancer stemness (55).

Understanding the role of the p53 as a regulator of stemness features in normal and malignant cells may lead to the identification of new therapeutic targets. Furthermore, enhanced cancer cell invasiveness, a key factor in metastasis and malignancy, is observed after down regulation of p53 (56). Some studies have demonstrated a correlation between p53 deficit and increased malignancy in some carcinomas with a de-differentiated phenotype, including HCC (57–59). p53 in response to a range of molecular changes in cells such as hypoxia, oncogene activation, and DNA damage induces cell cycle arrest and apoptosis. Activation of *TP53* not only ceases abnormal proliferation of cells, but also makes a commitment to a well-differentiated state (60). Conventional chemotherapy treatments destroy proliferative cancerous cells but not CSCs, which lead to relapse due to activation of CSCs. Therefore, combined with approved medical protocols, differentiation therapy with the induction of CSC-differentiation may help to prevent cancer relapse after therapy (57, 61).

As an appealing candidate for differentiation therapy, p53 has significant functions in various cell types with the restricting activity in the processes of reprogramming and dedifferentiation. Indeed, p53 has the potential to decrease self-renewal and induce differentiation in ESCs and cancer cells.The promotion of more differentiated progenies of CSCs is associated with pharmacological reactivation of *TP*53 (57, 61).

Mitophagy in HCC has been affected by mitochondrial dynamics through fission and fusion processes. Mitochondrial fission can induce cancer stem cell enrichment and stemness protection in liver cancer cells (62). The mitochondrial fission process can promote the intracellular accumulation of ROS, which leads to poor prognosis of HCC and the maintenance of CSC’s population, as well as a reduction in p53 activity (63).

**Figure 3:**
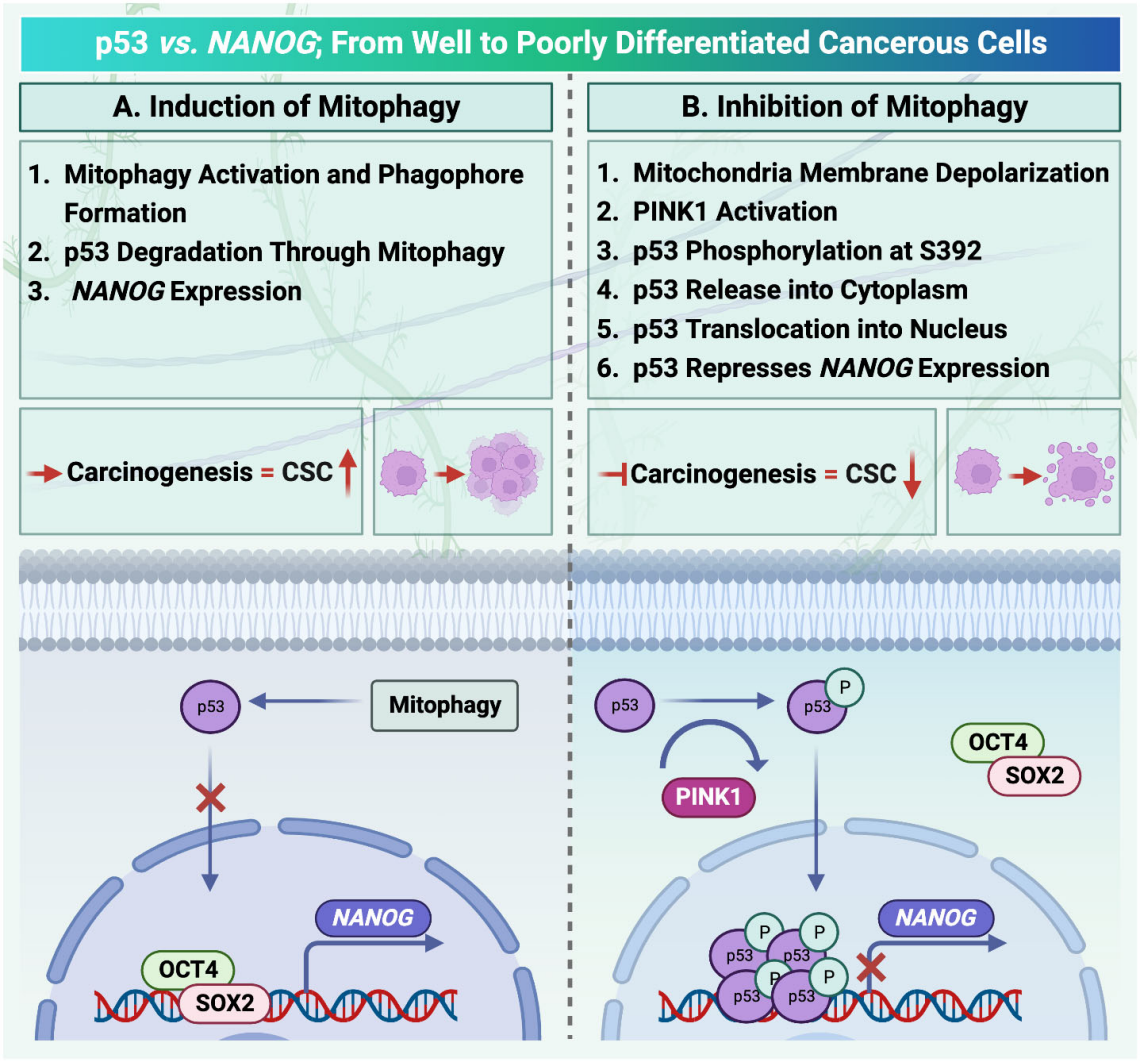
Mitophagy activation in hepatocellular carcinoma is under the control of p53/ checked by https://www.uniprot.org/. **(A)** Activation of mitophagy degrades cellular transcription factor p53. Therefore, there is no factor to compete with OCT4, SOX2 to locate on the NANOG promoter. **(B)** PINK1 can phosphorylate p53. Translocation of p53 increases after phosphorylation. As a result, the mitophagy activation process and NANOG expression are inhibited.

It has been reported that mitophagy can degrade and decrease cellular p53 (38). When mitophagy is inhibited, the mitophagy-associated kinase; PTEN-induced putative kinase 1 (PINK1), phosphorylates p53, which facilitates its nuclear translocation. [Nuclear p53 binds the *NANOG* promoter (**Figure 3**)] and suppresses its expression through interaction with OCT4 and SOX2 (55). As a result, the CSCs characteristics and their carcinogenesis capacity,their self-renewal and the maintenance of tumor propagation can be inhibited (64, 65). p53 mutations (mut p53)are essential for CSCs development and maintenance (66). Tumor gene mutation burden (TMB) is presented in **Figure 2C** to compare *TP53* mutation in poorly and well differentiated HCC cells. Poorly differentiated carcinomas have been found to have a high incidence of *TP53* mutations, which results in stem cell-like state transcriptomics (67). According to recent studies, wild-type p53 (wt p53) suppresses production of several liver CSC markers, including CD44, c-Myc, NANOG, SOX2 and OCT4.While p53 mutations would result in the absence of suppression on these CSC markers, and increased radio- and chemo-resistance (57, 68, 69).

Moreover, PINK1 is a serine/threonine protein kinase and required for mitophagy activation. The transcription levels of *PINK1*, in the liver, brain, breast, colorectal, esophageal, head and neck, and ovarian cancers, as well as leukemia and melanoma, have lower expression than normal tissues. However, in lymphoma, *PINK1* mRNA expression was reported to be markedly elevated (70).

According to the Human Protein Atlas’ analysis of *PINK1* expression by M. Wang et al. (2022) (70), *PINK1* may be either helpful or harmful with respect to the type of cancer. For instance, higher *PINK1* expression was linked to better overall survival in malignancies of the liver, kidney, pancreas, and endometrium, while it was associated with poor prognosis in breast, cervix, ovary, lung, glioblastoma, and melanoma cancers (70). For investigating the level of *PINK1* transcriptional expression in TCGA samples, 24 samples have been selected for analysis. Except for Skin Cutaneous Melanoma (SKCM), the expression of *PINK1* was lower in various types of tumors in comparison with normal tissues (**Figure 1B**).

Investigating the link between mitophagy and p53, the influence of PINK1 on p53 has shown that mitochondrial depolarization in HCC under stress conditionsby carbonyl cyanide chlorophenyl hydrazone (CCCP) results in enhanced mitophagy (71). As a result, recruiting PINK1on the mitochondrial outer membrane and phosphorylating p53 at S392 C-terminal entrapped it into mitochondria and then remove through the process of mitophagy (72). Therefore, NANOG expression and subsequently the number of liver CSCs can be increased. In contrast, when the inhibition of mitophagy is conducted by a fission inhibitor (Mdivi-1), the PINK1phosphorylated p53 can be translocated into the nucleus which suppresses *NANOG* promoter and results in liver CSCs population reduction (71). This finding suggested that PINK1 could be the elusive kinase that phosphorylates p53 at serine residue (73). PINK1 has also been identified as the first pro-autophagy molecule to be transcriptionally inhibited by p53 and it is revealed that PINK1 transcriptional repression needs just nucleus localization of p53.This is the first instance of an anti-autophagic phenotype being connected to the activity of nuclear p53 (70, 74). Furthermore, *TP53* transcript was demonstrated for an anti-mitophagic phenotype by inhibiting stemness features of cancerous cells and reducing chemoresistance (70, 75). Moreover, *TP53* inactivation decreased TIM23, TOM20, and HSPD1/HSP60 mitophagy indicators in cells as well as in the mouse brain while increasing expression of the autophagy receptors Optineurin (OPTN) and CALCOCO2/NDP52 and the production of LC3- II (75). It was also indicated that *TP53 *can be inactivated by pifithrin-α (PFT-α), a *TP53* transcription function blocker small molecule (76). The possible explanation of this phenomenon is that nuclear p53 can act as an anti-mitophagic factor by repressing *PINK1* transcription (77).

The cytoplasmic localization of p53 has been linked to its capacity to suppress autophagy. Transcriptional activity of p53 plays a role in the inhibition of autophagy (78).

Strategies to target p53 and recover the p53 pathway are entirely reliant on the p53 status, whether p53 is wild-type or mutant, in addition to the type of p53 mutation (79). To implement this strategy, some innovative modalities were considered, for instance *TP53*-based gene therapy, wild-type p53 stabilization, mutant p53 degradation (80), and restoration of function of a structural mutant to wild-type p53 by small molecules (81). In addition, the re-establishment of the p53 signalling cascade by activation of p53 downstream targets has been investigated (82).

Autophagy suppression can lead to an increase in p53 levels and its phosphorylation at serine 392 (S392), whereas autophagy stimulation has the reverse effect. When p53 is phosphorylated at position S392, it becomes activated and helps to localize the nucleus. These results thus demonstrated that autophagy can inhibit p53 activity (65).

In another study, when autophagy or mitophagy are inhibited, the induced p53 by PINK1was localized into the nucleus to suppress the expression of NANOG, a vital transcription factor for stem cells self-renewal (83).

Previously Mong-Lien Wang et al. underlined that NANOG along with OCT4, which has a POU domain, and SOX2, that has a high mobility group domain, are essential for maintaining pluripotency and self-renewal in undifferentiated embryonic stem cells (84).

*NANOG* is often overexpressed in hepatocellular carcinoma, particularly in cancer stem cells. There is a considerable association between the advanced level and poor prognosis of malignancy/aggressiveness and poorly differentiated HCC with *NANOG* expression (84). In addition, the overexpression of various downstream signaling involved in cancer initiation and progression, appears to originate from uncontrolled and abnormal *NANOG* expression. This appears to drive cells toward a reprogramming-like process but fails to maintain them on the path that leads to a normal stemness state. The ability of HCC cells to reproduce their lineage, give rise to differentiated cells, and communicate with their microenvironment in order to preserve a balance among quiescence, proliferation, and regeneration is known as the stemness state (85). CSCs exhibit stemness in a variety of features, including cancer progression and interaction with their microenvironment in quest of essential survival elements. As a result, CSCs can restore tumor mass post treatment (86).

Many molecular regulators, such as microRNAs, transcription factors, and kinases have been reported to mediate the silencing or overexpression of NANOG through post-transcriptional and translational regulation and thus regulate stemness and malignant transformation, as well as CSC-like phenotypes in cancer cells (50, 87, 88).

P53 is a transcription factor that is activated via stress and inhibits the growth of genetically damaged cells. Genomic instability following the loss or silencing of *TP53* promotes the clonal growth of abnormal cells. In the past decade, direct evidence for p53’s engagement in the stemness regulatory network has emerged, earning significant interest in the fields of cancer and stem cell research (89, 90).

P53-NANOG regulatory signals were shown to be involved in cancer cells, particularly in brain CSCs. NANOG is known to promote CSC-like features in primary p53-deficient adult mouse astrocytes, but not in astrocytes with intact p53. Interestingly, p53 is the gatekeeper, suppressing both cancer cells from further acquiring CSC properties as well as normal cells from tumor transformation (89, 90). The negative regulation of NANOG by p53 may contribute to its inhibitory effects on reprogramming and cancer stemness. Blocking NANOG and consequently, NANOG-mediated regulatory circuits, which account for approximately 50% of malignancies, might be a possible strategy to prevent tumor initiation and progression (89, 90).

A negative correlation between the amounts of NANOG and the levels of p53 and its S392 phosphorylated version has been revealed. Because of this, the connection between p53 and NANOG has been recognized in details and it was shown that phosphorylated p53 could bind to the NANOG promoter (73). Following this interaction, the transcription factors POU5F1/OCT4 and SOX2 are unable to bind to and activate the *NANOG* promoter, which suppresses the production of NANOG. These findings led researchers to conclude that, through limiting S392 phosphorylation and p53 activity, autophagy promotes NANOG expression and hepatic CSC proliferation (65).

As a response to hypoxia, NANOG binds to the *BNIP3L* promoter and induces mitophagy (91). However in another study, BNIP3L deficiency noticeably resulted in delayed/retarded tumor progression (92).

The levels of p53 and its S392 phosphorylated version are interestingly decreased and increased by the activation and inhibition of mitophagy, respectively (65). The S392 phosphorylated p53 was examined for its subcellular distribution, and it was found to be localized into the nucleus when mitophagy was blocked and to the mitochondria when it was triggered (65).

When the mitophagy process is initiated in the progression of cancer, the accumulation of phosphorylated p53 on the damaged mitochondria membrane can lead to removing p53 through the mitophagy process (65).

## Opinion perspective discussion

Mitophagy plays a crucial role in preserving homeostasis of cells and tissues. However, mitophagy is involved in tumorigenesis and cancer cell survival. p53 helps in the prevention of tumor growth by preserving a balance between self-renewal and differentiation of dividing cells. During carcinogenesis, p53 degrades through activated mitophagy. Therefore, NANOG can act as an oncogene to promote carcinogenesis by inducing cancer stem cells and mitophagy-associated HCC progression. However, when mitophagy is suppressed, p53 is phosphorylated on mitochondria by the mitophagy-related kinase PINK1, can be transferred into the nucleus so as to supress NANOG expression and prevent tumor progression.

Mitophagy acts as mitochondria quality control; however, the maintenance of CSCs population is increased by hepatocarcinogenesis. Chemotherapy resistance in CSCs is responsible for tumor progression and recurrence. Previous studies have sought whether the targeting of mitophagy signaling pathway can eradicate the cancer stem cell’s population in HCC. Therefore, the future direction in research might benefit from being focused on mitophagy targeting to diminish liver CSCs and keep the proper balance between p53 and NANOG. This will also provide a new opportunity for improving clinical trial outcomes and drug development studies in HCC.

## Author contributions

Conceptualizing, Writing – review & editing and designing of the manuscript have been done by MV, HM, and MH. The process of writing –drafting was conducted by FRN. YZ and NR involved in writing – drafting, Writing – review & editing and preparing figures.
